# Differences in 5-year weight change between younger and older US firefighters

**DOI:** 10.1186/s12889-021-11266-x

**Published:** 2021-06-24

**Authors:** Kevin C. Mathias, Yuchen Wu, Donald F. Stewart, Denise L. Smith

**Affiliations:** 1 Department of Health and Human Physiological Sciences, Skidmore College, 815 North Broadway, Saratoga Springs, NY 12866 USA; 2 Public Safety Occupational Health Center, 12099 Government Center Parkway, Fairfax, VA USA; 3 Illinois Fire Service Institute, University of Illinois at Urbana-Champaign, Champaign, IL USA

**Keywords:** Weight gain, Weight loss, Fat mass, Obesity, BMI

## Abstract

**Background:**

Research consistently finds that, on average, firefighters gain weight over time and some data indicate that younger firefighters are more likely to gain weight than older firefighters. The purpose of this study was to estimate the 5-year weight change among younger and older US firefighters.

**Methods:**

Data from two occupational medical exams separated by 5 years (2009–2016) were examined from a cohort of US career firefighters in Virginia (males, *n* = 589; females, *n* = 67). The cohort was grouped into two age categories (< 45 years and ≥ 45 years). Weight change subgroups were Loss (decrease of > 3% body weight), Stable (within ±3% body weight) and Gain (increase of > 3% body weight). Multinomial logistic regression models and linear regression models were conducted to examine differences in the probability of being in a particular weight change category, weight change overall and by weight change category between younger and older firefighters.

**Results:**

At baseline, 25 and 35% of younger (< 45 years) and older (≥ 45 years) were obese, respectively. Younger firefighters gained significantly (*P* < 0.05) more weight (3.0 ± 0.2 kg) than older firefighters (0.8 ± 0.5 kg). Younger firefighters were more likely to gain weight (53% versus 39%) and less likely (10% versus 20%) to lose weight as compared to older firefighters. Smaller weight gains were associated with age and BMI with the smallest increases observed in overweight and obese firefighters ≥45 years of age.

**Conclusions:**

Health care providers should be attentive to weight gain, even among young non-obese firefighters, and counsel firefighters to avoid the additive risks of being older and heavier. In addition, weight loss/management programs should be promoted in the fire service to encourage healthy body weight and to prevent unhealthy weight gain among both young and old firefighters alike.

## Introduction

Obesity is highly prevalent among US firefighters (24–35%) [1–5] and is associated with impaired work performance [6], increased risk of injury [7, 8] and duty-related sudden cardiac events [9]. Sudden cardiac events are the leading cause of duty-related deaths in the fire service [10], and coronary heart disease and structural heart changes (cardiomegaly and LVH) have been identified in over 80% of cardiac cases [11]. Obesity is related to adverse changes to the cardiovascular system, cardiovascular disease risk factors, and sudden cardiac death in both young and older firefighters [12–17]. Thus weight management is of significant concern for all firefighters, although little research has been conducted to understand how weight change differs between young and older firefighters.

Several longitudinal studies have shown that, on average, firefighters gain weight as they age (~ 0.5 kg/yr) [5, 18, 19] and that firefighters who gained weight over a 5-year period had adverse changes in cardiovascular health [20]. Previous studies, reporting on data from the 1980s and 1990s, suggests that while both age groups gain weight over time, younger firefighters gain more weight each year than older firefighters [18, 19]. Research has also shown that younger firefighters are less likely to receive weight loss advice, and young overweight and obese firefighters were less likely to receive weight loss advice as compared to their older counterparts [21]. Younger firefighters are also more likely to report having one or more barriers (e.g.,“lack of access to healthy foods” and “eating helps me cope with stress…”) to weight management [22]. Weight gain in older firefighters is also of particular concern, given that the risk of sudden cardiac death dramatically increases with age among firefighters [23]. The 10-year risk of a first atherosclerotic cardiovascular disease (ASCVD) event based on the pool-cohort equations [24] was 0.8 and 2.3% among normal versus obese 40–45 year old firefighters and 4.1 and 7.8% among normal versus obese ≥50 year old firefighters [25].

Previous studies reporting weight change over time in firefighters by age category were conducted around 25–40 years ago and the primary focus was not on younger versus older firefighters. In addition, previously reported average weight gain in firefighters failed to identify if excess weight gain in some individuals was masked by weight loss in others and the extent to which individuals gained or lost weight. Given the potential health risks associated with unhealthy weight gain among firefighters, the purpose of this study was to estimate the 5-year weight change (loss, stable, gained) among younger and older US firefighters. This information can be used to provide better insights for whether additional programming efforts are needed to encourage healthy weight management in younger and older firefighters.

## Methods

### Study population

Records from occupational medical exams that were performed between 2009 and 2016 were examined from a cohort of career firefighters in northern Virginia. The database included 1498 firefighters who had received at least one medical evaluation. The analytic sample was comprised of 656 firefighters (589 males and 67 females) who had two medical exam records (with body weight measured) separated by 5 years (range of 4–6 years) [20]. Data from the occupational medical exams were entered into a database by the occupational clinic staff. Informed consent was waived given that a de-identified dataset was transferred by the clinic to the First Responder Health and Safety Laboratory at Skidmore College. The Skidmore College Institutional Review Board reviewed and approved the study protocol.

### Data collection

An occupational health clinic that performs annual medical evaluations on a contract basis to a large county-wide fire department collected and provided data from the mandatory annual medical evaluations. Height and weight were recorded using a stadiometer and digital physician’s scale. Percent body fat was assessed via bioelectrical impedance (Tanita TBF-410) [26, 27]. Fat mass was calculated by multiplying bodyweight by the estimated percent body fat. Fat-free mass was calculated as bodyweight minus estimated fat mass.

### Definitions

Weight change categories were created based on a previously published cut-off to define weight maintenance [28]. The three categories of weight change were defined as Weight Loss (decrease > 3% in body weight); Weight Stable (change within ±3% in body weight); and Weight Gain (increase > 3% in body weight). Body mass index (BMI) classifications were based on cut points from the Centers for Disease Control and Prevention [29]. Firefighters were classified as Normal weight (< 25 kg/m^2^), Overweight (25–29.9 kg/m^2^), or Obese (≥30 kg/m^2^). Firefighters were grouped into two age categories Young (< 45 years; *n* = 509) and Old (≥45 years; *n* = 147) based in part on when age becomes a cardiovascular disease risk factor in men, to permit comparisons with previously published data [12, 13], and to ensure adequate sample sizes based on the mean age of the sample.

### Statistical analyses

All analyses were conducted using Stata 15.1 (StataCorp, College Station, TX, USA). Descriptive statistics were expressed as mean ± standard deviation or percent of subgroup in Table 1. The analysis of weight change was separated into two parts: the probability of being in a weight change category (lost, stable, gained) and the amount lost among firefighters within each weight change category. A multinomial logistic regression model was conducted with the three weight change categories as an outcome and age category as a categorical covariate. The post-estimation -margins- command was used to predict percentages within each weight change category for each age group and the total sample. The -dydx- command with –margins was used to test statistical differences between the age categories for the percentages within each weight change category. For weight change (kg), the point estimates and standard errors for each weight change category and the age categories were estimated using a linear regression model with covariates for age category, weight change category and a term for their interaction. The post-estimation -margins- command was used to predict weight change for each respective group. The -dydx- command within –margins- was used to test statistical differences between age categories for the change in weight within a weight change category. Separate linear regression models were conducted to estimate average weight change within each age group and average weight changed within each weight change category. Change in weight by baseline BMI category (normal, overweight, obese) and age category was first predicted using a linear regression model with categorical covariates for initial BMI category, age category and a term for their interaction. General trends analyses with a step-down approach were conducted with linear regression models starting with continuous terms for age, BMI and their interaction. The level of significance for all analyses was considered at *P* < 0.05 and was two sided for all tests.

**Table 1:** Firefighter cohort descriptive statistics at baseline by age category

	< 45 years old	≥45 years old
n	509	147
Duration Between Trials (y)	4.8 ± 0.6	4.7 ± 0.5
Age (y)	35.1 ± 6.0	48.5 ± 2.8
Male (%)	89	91
White/Other (%)	80	80
African American (%)	20	20
Height (m)	70.0 ± 3.1	69.8 ± 2.7
Weight (kg)	88.5 ± 14.6	89.9 ± 15.7
BMI (kg/m^2^)	27.8 ± 3.8	28.5 ± 4.1
Normal (%)	22	17
Overweight (%)	53	48
Obese (%)	25	35

Values are means ± SD or %

Normal weight (< 25 kg/m^2^), Overweight (25–29.9 kg/m^2^), or Obese (≥30 kg/m^2^)

## Results

**Fig. 1:**
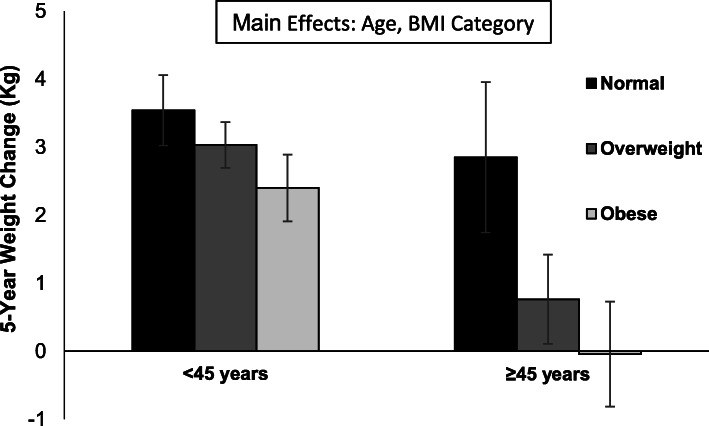
Weight change over 5 years among firefighters by age and BMI category. Values are means ± SE. Both age and Body Mass Index (BMI) (modelled as continuous variables) were independently and significantly (*P* < 0.05) associated with 5-year weight change. Normal weight (< 25 kg/m^2^), Overweight (25–29.9 kg/m^2^), or Obese (≥30 kg/m^2^)

The baseline characteristics of the cohort are presented in Table 1. At baseline, the average BMI was 27.8 and 28.5 kg/m^2^ for young and old firefighters and 25 and 35% of young and old firefighters were obese, respectively. Younger firefighters were more likely to gain weight (53% versus 39%) and less likely to lose weight (10% versus 20%) as compared to older firefighters (Table 2). On average, younger firefighters gained significantly more weight (*P* < 0.05) than older firefighters (3.0 ± 0.2 kg versus 0.8 ± 0.5 kg) (Table 3). Among those who gained weight, the increase was significantly higher (*P* < 0.05) among young (6.8 ± 0.2 kg) versus older (5.6 ± 0.4 kg) firefighters for the young and old firefighters, respectively. Significant differences within weight change categories (lost, stable, gained) between young and older firefighters were not found for fat mass or fat-free mass (Table 3). On average, younger firefighters gained fat mass over the 5-year period (1.0 ± 0.3 kg) whereas older firefighters lost fat mass (− 1.2 ± 0.5 kg). A significant difference in the change in fat free mass was not found between young and older firefighters across the 5-year period. Figure 1 presents weight change by baseline BMI category and age group. The general trends analyses suggested that both age and BMI were significant predictors of weight change; both older age and higher BMI were independently associated with lower weight gain over the 5-year period. On average, obese firefighters who were over 45 years did not have an increase in weight.

**Table 2:** Numbers and percentages of firefighters by age at baseline and weight change status over a 5-year period

	Lost Weight	Stable Weight	Gained Weight
< 45 years old	52 (10)	187 (37)	270 (53)
≥45 years old	29 (20)**	61 (41)	57 (39)**
Total Sample	81 (12)	248 (38)	327 (50)

Values are n (%)

Statistical comparisons between the age categories within each weight change category (lost, stable, gained) were conducted using a multinomial logistic regression model

** *P* < 0.01

**Table 3:** Change in body weight by age and weight change category over a 5-year period

	Lost Weight	Stable Weight	Gained Weight	Average of Category
Age Category	**Change in Body Weight ± SE (kg)**			
< 45 years old	− 7.4 ± 0.4	0.4 ± 0.2	6.8 ± 0.2	3.0 ± 0.2
≥45 years old	−6.7 ± 0.6	− 0.05 ± 0.4	5.6 ± 0.4*	0.8 ± 0.5***
Total Sample	−7.2 ± 0.3	0.3 ± 0.2	6.6 ± 0.2	2.5 ± 0.2
	**Change in Fat Mass ± SE (kg)**			
< 45 years old	−7.0 ± 0.8	−1.0 ± 0.4	3.6 ± 0.3	1.0 ± 0.3
≥45 years old	−7.7 ± 1.0	− 1.2 ± 0.7	2.0 ± 0.7	−1.2 ± 0.5**
Total Sample	−7.2 ± 0.6	−1.1 ± 0.4	3.4 ± 0.3	0.5 ± 0.2
	**Change in Fat-free Mass ± SE (kg)**			
< 45 years old	− 0.3 ± 0.7	1.5 ± 0.4	3.1 ± 0.3	2.2 ± 0.2
≥45 years old	0.9 ± 1.0	1.2 ± 0.6	3.7 ± 0.7	2.1 ± 0.4
Total Sample	0.1 ± 0.6	1.4 ± 0.3	3.2 ± 0.3	2.2 ± 0.2

Values are means ± SE

Statistical comparisons between the age categories within each weight change category (lost, stable, gained) were conducted using linear regression models. Separate linear regression models were conducted to generate the estimates for the total sample and the estimates for average of categories

*P < 0.05,**P < 0.01,***P < 0.001

## Discussion

The results of this study indicate that a large percentage of both young and old firefighters are obese. On average, both age groups gained weight, but the weight gain was not uniform for age categories or BMI categories. Younger firefighters were less likely to lose weight and more likely to gain weight than older firefighters which resulted in higher weight gain overall. Smaller weight gains were associated with higher age and BMI with the smallest increases observed in overweight and obese firefighters ≥45 years of age. Weight loss among both age categories was almost entirely fat mass, whereas, weight gain consisted of both fat mass and fat-free mass.

There is considerable evidence that as a group, firefighters gain weight over time; however, the weight gain is not uniform among individuals. Previous studies have shown that younger firefighters gain more weight than older firefighters. In a cohort of firefighters with baseline data from 1984 and followed for 7 years, average yearly increases in body weight of 0.73 kg, 0.53 kg, 0.41 kg, and 0.44 kg were found among the following age groups: 20–29 years, 30–39 years, 40–49 years, and 50–59 years, respectively [18]. Another cohort of firefighters with baseline data from 1996 found that younger firefighters (< 45 years old) gained roughly two times the weight per year (0.71 kg versus 0.34 kg) as compared to older firefighters (≥ 45 year olds) [19]. The current study found that while the weight change in younger firefighters is similar to previous studies, the weight gain among older firefighters (0.16 kg/year) is lower than past cohorts. Differences in weight change for older firefighters could be due to cohort effects, more guidance from their physicians, or greater adoption of recent health promotion programs initiated within the fire service [30–32].

This study improves upon previous research on age and weight gain in firefighters by examining both positive and negative weight changes in addition to the overall change. Younger firefighters in this cohort were half as likely to lose weight as older firefighters (10% versus 20%), even though both young and old firefighters had mean BMIs in the overweight range (28.0 kg/m^2^ and 28.6 kg/m^2^, respectively) at baseline. The similar percentage of normal weight firefighters in younger and older firefighter groups (22 and 17%) indicates that the lower likelihood of weight loss observed in the younger firefighters was not primarily due to fewer individuals who had excess body weight.

There are several potential explanations for larger weight gains among younger firefighters. Younger firefighters could have more barriers (e.g.,“lack of access to healthy foods” and “eating helps me cope with stress…”) to weight management [22]. Younger firefighters are also less likely to receive weight loss advice from primary care physicians [21], although the positive impact of advice on weight change in firefighters is inconclusive [33, 34]. Furthermore, younger adults in the general population are also more likely to gain weight than older adults [35], which indicates that firefighters are not an exception to a general tendency of weight gain with age in the United States. It is also possible that excess weight may not be perceived as presenting the same health risk in younger versus older firefighters, and possible that this perception is shared by both firefighters and their health care providers.

In the general population, overweight young adults are more likely to gain additional weight over time than young adults with heathier body weights [36]. Previous studies in firefighters have found mixed results with respect to weight gain between those with heathier versus less healthy body weight [18, 19]. In the current study, a higher BMI was significantly associated with smaller weight gains over the 5-year period and smallest in overweight and obese firefighters ≥45 year of age. These findings may indicate that weight maintenance efforts were partially effective in older firefighters with excess body weight; however, given the high prevalence of overweight and obesity and the detrimental association between obesity and cardiovascular health, further weight loss is needed. It is important to note that the weight loss observed in both young and older firefighters was estimated to be primarily body fat, and could indicate that lean tissue was not lost during the process of weight loss. The findings also found that the majority of weight gain was due to increases in fat mass, and this same cohort of firefighters with weight gain was also shown to have an increased prevalence of cardiovascular disease risk factors over the 5-year period [20].

The primary strengths of this study were that weight loss and weight maintenance were measured in addition to weight gain. This study also examined the nature of the weight change by estimating change in fat mass and fat free mass. An additional strength was that the occupational medical exams that were used to obtain data were mandatory which minimized selection bias. A limitation of this study was that body weights were obtained as part of a medical evaluation and precise measurements of weight may not have been obtained. However, given that all of the weight measurements were obtained from a single clinic, and were obtained in the same manner for both young and old firefighters, measurement error was unlikely to alter the interpretation of the results. Our study relied on a single cut-off for defining young and old. The age cut off was chosen based on dividing the sample into two groups that would generally be considered “young” and “old” based on common definition and published literature, which in part reflects when age becomes a risk factor for CVD in men. We recognize age becomes a risk factor for men and women at different ages (45 and 55 years, respectively), and future research on weight change stratified by sex and across the age continuum would provide further insight into this issue. Finally, this study relied on data from a single fire department; therefore, the results may not reflect weight change among firefighters at the national level.

Given the high prevalence of obesity in the fire service and association with a higher risk of on-duty injury and sudden cardiac death, further efforts are needed to address weight gain among young and old firefighters alike. While older firefighters, particularly those with excess body weight are at higher risk of a cardiovascular event, the results of this study indicate that efforts to prevent younger firefighters from becoming at-risk individuals at older ages through continual unhealthy weight gain are either lacking or ineffective. Health care providers who evaluate firefighters should be attentive to the tendency for weight gain, and counsel firefighters on the need to avoid the additive risks of being older and heavier with respect to job performance, the risk of sudden cardiac arrest, and overall health. Further, our findings suggest that effective weight loss/management programs are needed in the fire service to encourage healthy body weight and to prevent unhealthy weight gain among both young and older firefighters.

## Data Availability

The datasets generated and analyzed during the current study are not publicly available and were provided by an occupational health clinic under the agreement that the data would only be viewed by the First Responders Health and Safety Laboratory and used for publication purposes. Requests for further information on the data or analysis/results should be directed to the corresponding author.

## References

[1] Soteriades ES, Hauser R, Kawachi I, Christiani DC, Kales SN (2008). Obesity and risk of job disability in male firefighters. Occup Med (Lond).

[2] Choi B, Steiss D, Garcia-Rivas J, Kojaku S, Schnall P, Dobson M, Baker D (2016). Comparison of body mass index with waist circumference and skinfold-based percent body fat in firefighters: adiposity classification and associations with cardiovascular disease risk factors. Int Arch Occup Environ Health.

[3] Poston WS, Haddock C, Jahnke S, Jitnarin N, Tuley B, Kales S (2011). The prevalence of overweight, obesity, and substandard fitness in a population-based firefighter cohort. J Occup Environ Med.

[4] Smith DL, Fehling PC, Frisch A, Haller JM, Winke M, Dailey MW (2012). The prevalence of cardiovascular disease risk factors and obesity in firefighters. J Obes.

[5] Smith DL, Graham E, Stewart DF, Mathias KC (2020). Cardiovascular disease risk factor changes over 5 years among male and female US firefighters. J Occup Environ Med.

[6] Kesler RM, Ensari I, Bollaert RE, Motl RW, Hsiao-Wecksler ET, Rosengren KS, Fernhall B, Smith DL, Horn GP (2018). Physiological response to firefighting activities of various work cycles using extended duration and prototype SCBA. Ergonomics..

[7] Poston WS, Jitnarin N, Haddock CK, Jahnke SA, Tuley BC (2011). Obesity and injury-related absenteeism in a population-based firefighter cohort. Obesity (Silver Spring).

[8] Jahnke S, Poston W, Haddock C, Jitnarin N (2013). Obesity and incident injury among career firefighters in the Central United States. Obesity..

[9] Kales SN, Soteriades ES, Christoudias SG, Christiani DC (2003). Firefighters and on-duty deaths from coronary heart disease: a case control study. Environ Health.

[10] Fahy RF, Petrillo JT, Moliz JL (2020). Firefighter fatalities in the United States – 2019.

[11] Smith DL, Haller JM, Korre M, Fehling PC, Sampani K (2018). Pathoanatomic findings associated with duty-related cardiac death in US firefighters: a case-control study. J Am Heart Assoc.

[12] Yang J, Teehan D, Farioli A, Baur DM, Smith D, Kales SN (2013). Sudden cardiac death among firefighters ≤45 years of age in the United States. Am J Cardiol.

[13] Farioli A, Yang J, Teehan D, Baur DM, Smith DL, Kales SN (2014). Duty-related risk of sudden cardiac death among young US firefighters. Occup Med (Lond).

[14] Fahs CA, Smith DL, Horn GP, Agiovlasitis S, Rossow LM, Echols G, Heffernan KS, Fernhall B (2009). Impact of excess body weight on arterial structure, function, and blood pressure in firefighters. Am J Cardiol.

[15] Soteriades ES, Smith DL, Tsismenakis AJ, Baur DM, Kales SN (2011). Cardiovascular disease in US firefighters: a systematic review. Cardiol Rev.

[16] Korre M, Porto LGG, Farioli A, Yang J, Christiani DC, Christophi CA, Lombardi DA, Kovacs RJ, Mastouri R, Abbasi S, Steigner M, Moffatt S, Smith D, Kales SN (2016). Effect of body mass index on left ventricular mass in career male firefighters. Am J Cardiol.

[17] Bode ED, Mathias KC, Stewart D, Moffatt SM, Jack K, Smith DL. Cardiovascular disease risk factors by BMI and age in United States firefighters.Obesity (Silver Spring). 2021;29(7):1186-94. 10.1002/oby.23175. 10.1002/oby.23175 PMC8362202 34060241

[18] Gerace TA, George VA (1996). Predictors of weight increases over 7 years in fire fighters and paramedics. Prev Med.

[19] Soteriades ES, Hauser R, Kawachi I, Liarokapis D, Christiani DC, Kales SN (2005). Obesity and cardiovascular disease risk factors in firefighters: a prospective cohort study. Obes Res.

[20] Mathias KC, Bode ED, Stewart DF, Smith DL (2020). Changes in firefighter weight and cardiovascular disease risk factors over five years. Med Sci Sports Exerc.

[21] Wilkinson ML, Brown AL, Poston WS, Haddock CK, Jahnke SA, Day RS (2014). Physician weight recommendations for overweight and obese firefighters, United States, 2011–2012. Prev Chronic Dis.

[22] Muegge CM, Zollinger TW, Song Y, Wessel J, Monahan PO, Moffatt SM (2020). Barriers to weight management among overweight and obese firefighters. J Occup Environ Med.

[23] Farioli A, Christophi CA, Quarta CC, Kales SN (2015). Incidence of sudden cardiac death in a young active population. J Am Heart Assoc.

[24] Goff DC, Lloyd-Jones DM, Bennett G, Coady S, D'Agostino R, Gibbons R (2014). 2013 ACC/AHA guideline on the assessment of cardiovascular risk: a report of the American College of Cardiology/American Heart Association task force on practice guidelines. Circulation..

[25] Hollerbach BS, Mathias KC, Stewart D, Jack K, Smith DL (2020). A cross-sectional examination of 10-year atherosclerotic cardiovascular disease risk among US firefighters by age and weight status. J Occup Environ Med.

[26] Kyle UG, Bosaeus I, Lorenzo ADD, Deurenberg P, Elia M, Gomez JM (2004). Bioelectrical impedance analysis-part II: utilization in clinical practice. Clin Nutr.

[27] Vasold K, Parks AC, Phelan DM, Pontifex MB, Pivarnik J (2019). Reliability and validity of commercially available low-cost bioelectrical impedance analysis. Int J Sport Nutr Exerc Metab.

[28] Stevens J, Truesdale KP, McClain JE, Cai J (2006). The definition of weight maintenance. Int J Obes.

[29] Centers for Disease Control and Prevention (2020). Defining adult overweight and obesity.

[30] 2018 National Volunteer Fire Council (2021). Heart healthy firefighter program.

[31] International Association of Fire Fighters and International Association of Fire Chiefs (2018). The fire service joint labor management wellness fitness initiative: 4th edition.

[32] Technical Committee on Fire Service Occupational Safety and Health. NFPA 1582: standard on comprehensive occupational medical program for fire departments. National Fire Protection Association. 2018. https://www.nfpa.org/codes-and-standards/all-codes-and-standards/list-of-codes-and-standards/detail?code=1582. Accessed July 2020.

[33] Brown AL, Poston WSC, Jahnke SA, Haddock CK, Luo S, Delclos GL (2016). Weight loss advice and prospective weight change among overweight firefighters. Int J Occup Environ Health.

[34] Brown AL, Poston WS, Jahnke SA, Haddock CK, Luo S, Delclos GL (2015). Weight advice associated with male firefighter weight perception and behavior. Am J Prev Med.

[35] Lewis CE, Jacobs DR, McCreath H, Kiefe CI, Schreiner PJ, Smith DE (2000). Weight gain continues in the 1990s: 10-year trends in weight and overweight from the CARDIA study. Coronary artery risk development in young adults. Am J Epidemiol.

[36] Truesdale KP, Stevens J, Lewis CE, Schreiner PJ, Loria CM, Cai J (2006). Changes in risk factors for cardiovascular disease by baseline weight status in young adults who maintain or gain weight over 15 years: the CARDIA study. Int J Obes (Lond).

